# The Impact of Fintech Development on Air Pollution

**DOI:** 10.3390/ijerph20043387

**Published:** 2023-02-15

**Authors:** Yuzhen Ma, Xinyang Wei, Gaoyun Yan, Xiaoyu He

**Affiliations:** 1 School of Business, Macau University of Science and Technology, Taipa, Macau 999078, China; 2 Business School, Nagoya University of Commerce and Business, 4-4 Sagamine, Komenoki-cho, Nisshin, Aichi, Nagoya 470-0193, Japan; 3 ARC Centre of Excellence in Population Ageing Research, University of New South Wales, Sydney, NSW 2052, Australia

**Keywords:** Fintech, air pollution, SO_2_, PM_2.5_, dust

## Abstract

Over the past 40 years of reform and opening-up, China has achieved rapid economic and technological growth at the cost of severe air pollution. The emerging Fintech, as the result of financial institutions’ adapting to the latest digital technology, might be a solution to reduce air pollution. This paper investigates the impact of Fintech development on air pollution using a two-factor fixed effects model based on data for prefecture-level cities in China from 2011 to 2017. The findings show that Fintech development can effectively reduce air pollution emissions, and this conclusion is proved to be robust throughout a series of tests. The mechanism analysis shows that Fintech reduces air pollution by promoting digital finance and green innovation.

## 1. Introduction

Air pollution is a complex mixture of gaseous and particulate components that varies in space and time [1]. Since rapid industrialization, air pollution has become one of the most important environmental issues worldwide. Some scholars have found that a larger economy could increase air pollution while keeping related environmental protection policies unchanged [2,3,4]. Other scholars have found that some air pollutants have negative effects on human health [5,6,7]. Therefore, the synergy between the environment and the economy [8] and the impact of the environment on people’s health [6] are the key questions that need to be addressed by governments worldwide.

China’s economic growth largely depended on fossil fuels [9]. Rapid urbanization and industrialization have accelerated urban air pollution [10]. In response to that, China enacted the Law on the Prevention and Control of Atmospheric Pollution Air (Air Pollution Law henceforth) in 1987. The government further amended the Air Pollution Law in 2000, requiring the installation of flue gas desulphurization technology systems. Moreover, air pollution control was set as a mandatory goal in the 11th Five-Year Plan, and a national mandate to control NO_X_ emissions was established in the 12th Five-Year Plan. In addition, more stringent control of PM_2.5_ emissions was introduced by the 2013–2017 Air Pollution Prevention and Control Action Plan issued by the State Council [9]. The authors of [8] examined China’s control of air pollution and suggested collaborative government intervention and further development of innovative technologies. In September 2022, China’s Ministry of Ecology and Environment issued a special 14th Five-Year plan for science and technology innovation in the area of ecology and environment. This plan requested more research targeting major environmental issues to develop key technologies for pollution prevention and control, environmental monitoring, and risk control. The plan also accelerated the development of a green technology bank to promote green technology innovation and financial services. Therefore, this policy provides strong motivations to advance new technologies such as big data, cloud computing, and artificial intelligence and to promote interdisciplinary integration.

Fintech was earlier explained in [11] as an acronym for financial technology that combines banking expertise, modern management science technology, and computers. Fintech has been closely studied in financial economics and business behavior. The authors of [12] showed that Fintech is a combination of finance and technology: as technology has always had a significant impact on the financial industry, technological advancements have changed the way the financial industry operates. The financial industry, as a result, has been characterized by technology throughout its history [13]. Due to the rapid development of the Internet, information technology, mobile phones, and other digital technologies [14], Fintech has expanded dramatically in the financial sector. For example, IT costs account for 15–20% of the total costs in the banking industry and have become the second largest cost factor following labor costs [15]. Moreover, banks have the highest IT investments among all industries, which are between 4.7 and 9.4% of their revenues, while insurance companies and airlines have IT investments of 3.3% and 2.6% of their revenues, respectively [16]. Fintech simplifies the bank lending process [17], causing it to be easier for SMEs to obtain loans [18], whereas P2P directly connects both lenders and borrowers, causing banks to no longer be needed as the middlemen between borrowers and lenders [19,20]. Therefore, the development of Fintech will affect the liquidity of traditional banking deposits and impact economic development [21]. Fintech, third-party payment, credit, and insurance significantly boost China’s economic growth, and there is a two-way causal relationship between Fintech and economic growth [14]. Some scholars have demonstrated the importance of Fintech in economic growth regarding financial resource aggregation, financial efficiency, and regional innovation levels [22,23,24].

Extending the literature to environmental pollution and Fintech, we notice that most studies have shown that Fintech can reduce environmental pollution and promote sustainable development [25,26,27]. In terms of greenhouse gases, some researchers have found that the development of Fintech can significantly reduce carbon dioxide emissions [28,29,30]. However, to date, little is known about the green attributes of Fintech [25].

Therefore, this study aims to fill the gap and investigate the impact of Fintech development on air pollution using prefecture-level city data in China from 2011 to 2017. Our results from the fixed effect model confirm that the development of Fintech can significantly reduce air pollution. The mechanism analysis shows that Fintech mainly reduces air pollution by promoting digital finance and enhancing the city’s green innovation capabilities.

This paper presents several contributions to the literature. First, unlike most studies to date that use digital finance as the measure of Fintech, this paper directly evaluates the impact of Fintech on air pollution and selects digital finance as one possible mechanism (note that Fintech broadly refers to the application of technological innovation in the field of financial business, such as the use of cloud computing, big data, and other emerging technologies to collect users’ information to build a default database and apply it to the financial industry, while digital finance broadly refers to the internationalization of offline business, that is, the business model of traditional financial institutions and new providers using digital technology in the delivery of financial services, such as online large loans and online money transfer. Overall, Fintech focuses more on finance, developing new science and technology and applying them to finance, while digital finance, on the other hand, focuses more on the frontier of digitalization, serving financial services through the accumulation, analysis, and judgment of financial data). We believe that Fintech and digital finance are not equal despite the common ground of technology and finance. Therefore, this study broadens the research of Fintech and environmental pollution. Second, to our knowledge, this paper is the first one choosing digital finance and green innovation capabilities as the research objects to explore the mechanism of Fintech development on air quality. This study also confirms that digital finance and green innovation capabilities are two crucial mechanisms. Third, this paper provides a more comprehensive discussion with multiple alternative measures of the outcome variables in the robustness tests.

In this paper, we discuss the impact of the development of Fintech on air pollution, specifically through the study of two mechanisms, namely the digital financial mechanism and the green innovation mechanism.

In terms of digital financial mechanisms, Fintech can contribute to the development of the digital economy through multiple channels [31]. The authors of [32] suggest that Fintech is conducive to expanding the scope of a trade by increasing long-tail consumption, investment conversion, and credit capital accumulation, thereby promoting the growth of the digital economy. On the other hand, the authors of [33] believe that Fintech’s contribution is mainly from the creation effect, information effect, inclusive effect, long-tail effect, and security. The five aspects of the effect can promote the development of the digital economy. Digital finance is a branch of the digital economy [34]. The impact of Fintech on the digital economy will empower digital finance. Regarding digital finance and air pollution, digital finance can reduce pollution, while the entrepreneurial effect, innovation effect, and industrial upgrading effect are important mechanisms [35]. The authors of [36] use the GMM estimation method to analyze provincial panel data and find that the development of digital finance can help reduce pollution emissions. This emission reduction effect is mainly achieved by increasing the coverage and depth of digital finance. The development level of digital finance has a significant effect on green technology innovation in heavily polluting industries. The development level of digital finance can promote green technology innovation in heavily polluting industries and reduce pollution emissions by alleviating the financing constraints of these industries [37].

Digital finance can reduce air pollution by promoting industrial structure upgrade [38] and achieving a more rational resource allocation [39,40,41]. Specifically, the development of digital finance can provide sufficient financial support for enterprises to reduce emissions. For instance, the authors of [42] use data from China’s A-share listed companies from 2011 to 2018. The authors find that digital finance has eased the financing constraints of listed companies and improved their overall innovation capabilities, resulting in a lower pollution level due to companies’ enhanced green innovation capabilities. Similar effects are also found with Fintech. The authors of [43] suggest that Fintech can significantly improve the green technology innovation capabilities of enterprises. The authors of [44] reached the same conclusion: in cities with more developed financial technology, enterprises have obtained more green patents and have higher innovation efficiency. Furthermore, the findings in [45] suggest that Fintech promotes green innovation by providing businesses with a better supply of capital and stronger financial support.

Green innovation is an effective way to reduce pollution. The effectiveness appears in slowing down pollutant emissions and purifying and absorbing the existing pollutants, thus bringing beneficial environmental effects [46]. The authors of [47] use the system generalized method of moments (SYS-GMM) to analyze the cross-sectional data of 30 provinces in China from 1997 to 2017. The study finds that green innovation has a positive effect on the reduction in sulphur dioxide SO_2_. In addition, green technology innovation has positive spillover effects on controlling smog pollution [48]. Similarly, green innovation also has a promising impact on greenhouse gas emissions. The authors of [49]’s results from using the STIRPAT model confirm that improving green innovation capabilities can significantly reduce carbon dioxide emissions.

Therefore, in this paper, we propose two hypotheses as follows:

**Hypothesis 1:**  *Fintech development can effectively reduce air pollution*.

**Hypothesis 2:**  *Fintech can reduce air pollution by promoting green innovation and digital finance development*.

The first part of this paper introduces the current research status of Fintech and air pollution before describing the two mechanisms selected and the hypotheses proposed in this study. The second part introduces the data source and variable information, while the third part presents the benchmark model and replacement for the robustness check. The last part concludes with the corresponding policy recommendations.

## 2. Materials and Methods

### 2.1. Data

This study uses data from multiple sources, including the China City Statistical Yearbook, internet search results by using crawler technology, and publications from Peking University and the China National Intellectual Property Administration. In terms of the sample, we target prefecture-level cities in China from 2011 to 2017.

Our outcome variables are the city air pollution data from the China City Statistical Yearbook, namely SO_2_, PM_2.5_, and dust. Due to the large scale in values, we adopt the natural logarithm for the SO_2_ emissions and dust, while the index of PM_2.5_ is the yearly average value for each city.

The key variable of interest, Fintech, is a city-level annual index calculated by the natural logarithm of the total number of search results for a city on Baidu News each year (Baidu is a search engine that is broadly used in China, also known as the Chinese equivalent of Google). For each city, the keywords for the Baidu News search are set in the format of “city name + a Fintech-related keyword”. Specifically, 48 Fintech-related keywords are used for all the cities in our data, such as Internet finance, machine learning, cloud computing, quantitative finance, big data, etc. Therefore, the Fintech variable constructed in this paper will reflect the level of Fintech development in the city between 2011 and 2017. The logic of using Baidu News search results to measure the level of Fintech development is that the development of Fintech and technological innovation in a region is often the focus of media, so it can be retrieved by the news search engine. As a leading Chinese search engine, Baidu has an absolute monopoly in the Chinese search engine market. Therefore, the number of search results obtained from Baidu News search for fintech-related keywords can reflect the development level of fintech in the relevant regions [50].

We also obtain other city-level information from the China City Statistical Yearbook, such as foreign direct investment (FDI), green coverage, urbanization level, number of university students, and regional GDP. Specifically, green coverage is the green coverage rate of the built-up area of the municipal area. Urbanization is defined as the municipal district’s land area divided by the prefecture-level city’s land area, indicating the urbanization level of a city. Among these control variables, the natural logarithm is adopted for the city-level FDI and the number of university students.

Moreover, two more indices are included in this study to investigate the mechanisms through which Fintech can reduce air pollution, i.e., digital finance and green patent. The digital finance data are from the Digital Financial Inclusion Index of Peking University, while the green innovation data are the annual number of green patents for each city from the China National Intellectual Property Administration. We use the natural logarithm of the green patent number for the model estimation. After deleting observations with missing values, 226 cities remained in the sample.

### 2.2. Methodology

We employ a two-factor fixed effects model to estimate the impact of Fintech on air pollution, allowing for city-fixed effect and time (year)-fixed effect. Robust standard errors are set to cluster at the city level. The specific model is as follows:

(1) $$ln{\mathrm{SO}}_{2}{}_{ct}=c+\beta Fintech_{ct}+X_{ct}^{\prime }\gamma +\mu_{c}+\mu_{t}+\varepsilon_{ct}\;$$

where *c* is the constant term; the dependent variable $lnSO_{2}{}_{ct}$ is the natural logarithm of SO_2_ emissions of city *c* in year *t*; $Fintech_{ct}$ is the Fintech index representing city *c*’s Fintech level in year *t*; $X_{ct}$ contains a set of city-level control variables, including GDP, FDI, number of university students, urbanization, and green coverage; $\mu_{c}$ is the city-fixed effect; and $\mu_{t}$ is the time (year)-fixed effect.

The detailed variable definition and descriptive statistics are shown in Table 1. During the sample period, the mean value of the sulfur dioxide emission is 10.41, with the maximum value being 13.18, indicating that most of the regions in the sample have heavy sulfur dioxide emissions and relatively low air quality. The Fintech variable in our sample has a mean of 2.70, indicating an average of 14.94 Baidu news keyword search results during the sample period. In addition, for the control variables, it is worth noting that for the city-level covariates such as green coverage, urbanization, and FDI, the maximum and the minimum values differ significantly, suggesting remarkable variation in the level of development across the sample cities in China.

**Figure 1:**
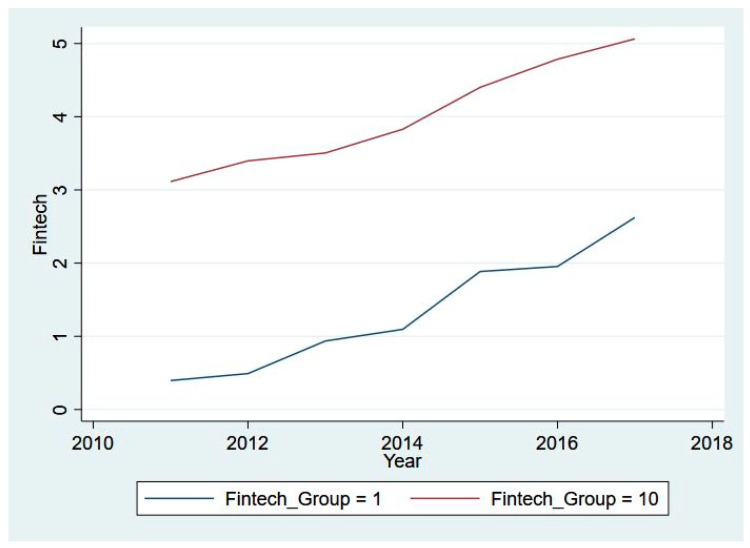
Fintech development for 1st and 10th groups, 2011–2017.

**Figure 2:**
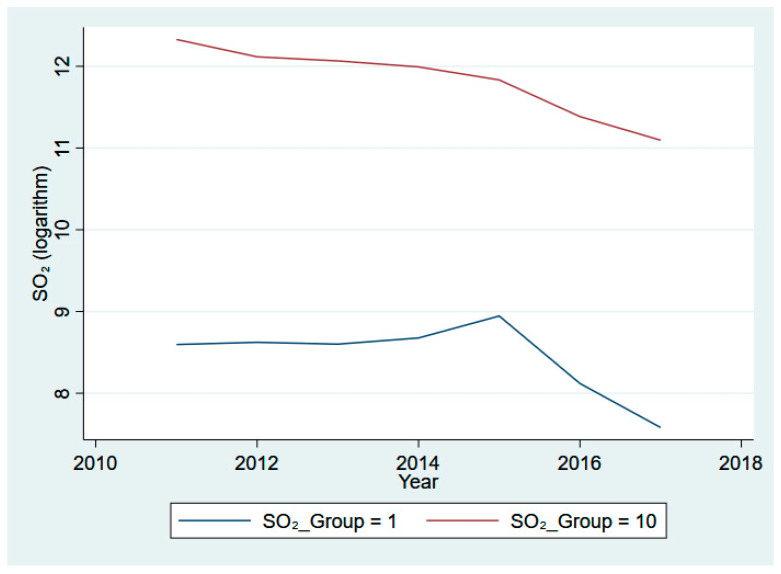
SO_2_ emissions for 1st and 10th groups, 2011–2017.

**Figure 3:**
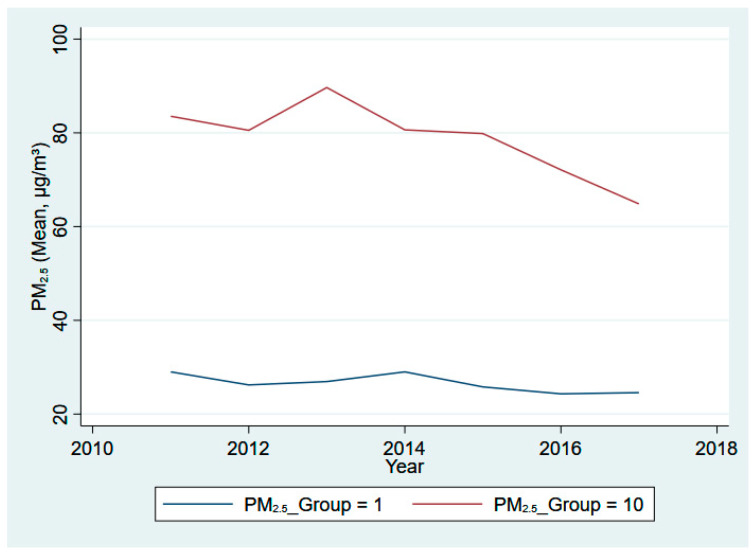
PM_2.5_ emissions for 1st and 10th groups, 2011–2017.

**Figure 4:**
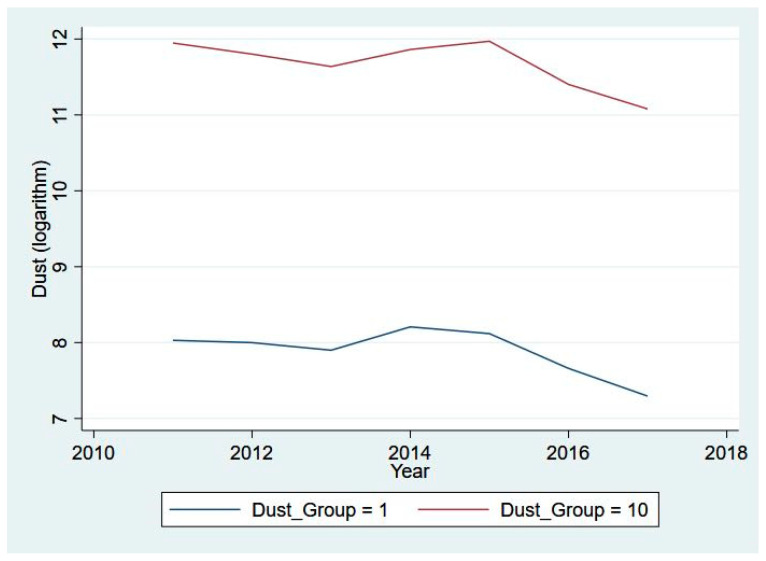
Dust level for 1st and 10th groups, 2011–2017.

Moreover, we illustrate the clustered mean of our core variables across different decile groups (the 1st decile group in blue and the 10th decile group in red) from 2011 to 2017 in Figure 1, Figure 2, Figure 3 and Figure 4. Specifically, Figure 1 presents the eight-year trend of Fintech development, while the trends of pollutant emission levels are displayed in Figure 2, Figure 3 and Figure 4. Figure 1 shows a rising trend in Fintech development over the sample period. As expected, the level of three types of air pollutants (SO_2_, PM_2.5_, and dust) share a common overall decreasing trend from 2011 to 2017, despite some differences in fluctuation and scale of drop.

**Figure 5:**
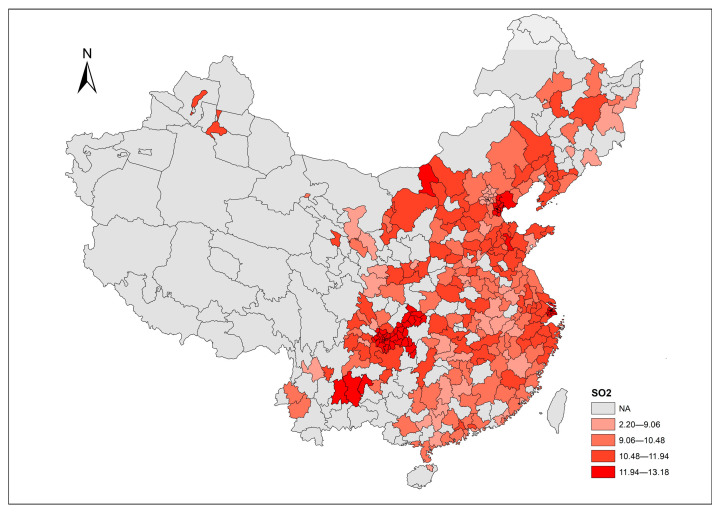
SO_2_ emission across cities in 2017.

**Figure 6:**
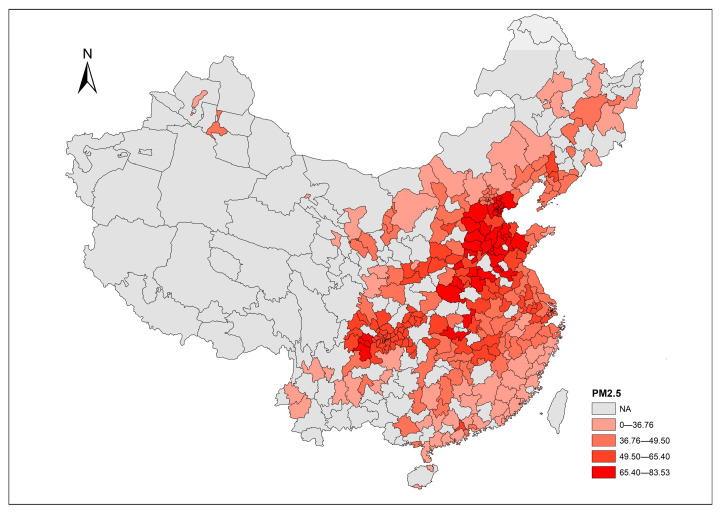
PM_2.5_ emission across cities in 2017.

**Figure 7:**
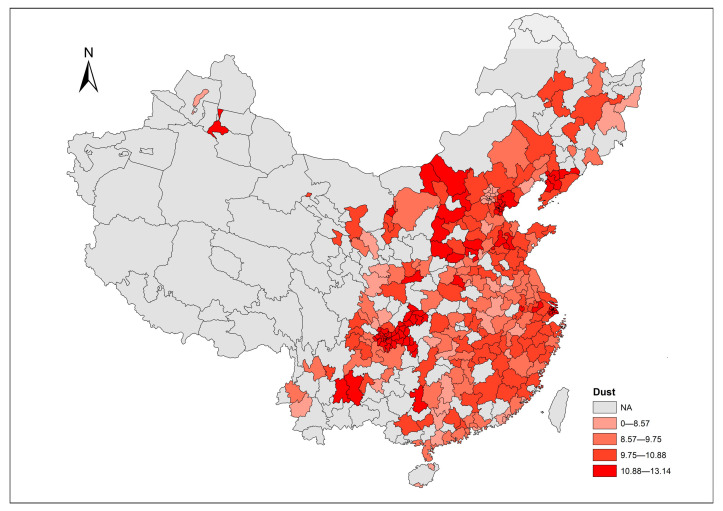
Dust level across cities in 2017.

In addition to the time trend, the spatial distributions of each air pollutant provide a straightforward overview across cities. The corresponding distributions of air pollutants in 2017 are mapped using ArcGIS 10.2 software (Figure 5, Figure 6 and Figure 7), with values divided into five levels according to the Jenks natural breaks classification. In the figures, grey areas are the regions where data are unavailable. For our sample cities, the darker color represents a higher level of air pollutant emissions. Relative to PM_2.5_, it seems that SO_2_ and dust are the more common issues in terms of most of our sample cities in 2017. Figure 5 shows that the emission of SO_2_ in the central-western region is moderately higher than other regions in China. Figure 6 highlights the severity of PM_2.5_ emission distributed in a diagonal line from north China to central China. The distribution of dust levels in the sample cities in Figure 7 reveals a wide range of cities suffering from dust problems in 2017.

## 3. Results and Discussion

### 3.1. Basic Regression Model

A stepwise regression method is applied for the main specification, with the results presented in Table 2. Columns (1)–(6) show the six models with gradually added covariants, respectively. As mentioned earlier, the city-fixed effect and time (year)-fixed effect are included in all models, while the robust standard errors are clustered at the city level.

Column (1) presents the results of a baseline model with only Fintech as the explanatory variable, along with fixed effects. Other city-level explanatory variables are gradually added to the models from Column (2) to Column (6), such as green coverage, FDI, urbanization, number of university students, and GDP. Column (6) shows the results of the model with the complete set of covariants.

The estimate results in Column (1) show a significant negative effect of the Fintech development on industrial SO_2_ emissions, implying that the higher the degree of Fintech development for a city, the greater the emission reduction effect of the city’s SO_2_ emissions.

This result is consistent across all six models, despite the variation in magnitude. As expected, in line with the other included explanatory variables, the size of the negative effect of Fintech on air pollution is decreasing, although the effect remains significant. The results in Column (6) confirm that the development of Fintech still has a significant and promising impact on the reduction in SO_2_ emission after adding the complete set of control variables. Specifically, every 1 per cent increase in the Fintech development leads to a 0.07 per cent drop in SO_2_ emissions. Therefore, research hypothesis 1 of this paper has been verified.

We propose two possible channels through which the development of Fintech may affect the local air pollution level. First of all, technological changes in the financial industry, especially digital finance, have caused it to be easier for companies to raise funds. Traditionally, access to capital from the financial sector relies on the financial data of a business that can demonstrate profitability and low risks. As far as the risks and uncertainties of enterprises’ R&D are concerned, such a method is difficult to provide effective financing for technological innovation enterprises. Fintech, such as crowdfunding, causes it to be easier for enterprises, especially small and micro enterprises and start-ups, to obtain financing through the advantages of “openness, a small amount, and public” [24]. As air pollution control in China becomes increasingly mandatory [9] and industries need to seek reform and innovation, easy access to capital may become even more necessary. Second, Fintech can promote green finance, provide green financial services for more polluting companies, and guide them to green transformation, thus strengthening sustainable development [25].

Among the control variables, the coefficient of FDI is significant and negative, indicating that the “pollution halo” effect of foreign investment is prominent, and FDI can improve the environmental quality of the host country through technology spillover effects [51,52,53]. The number of university students is positively correlated with SO_2_ emissions according to the results. We argue that this positive effect may be because the distribution of universities in China is heavily concentrated in more developed cities with larger populations. Therefore, cities with more university students are also larger cities that may consume more energy, thus increasing air pollution emissions [54].

### 3.2. Robust Tests

To ensure that the main results are robust across different air pollution measures, we use two alternative outcome variables to replace SO_2_ in the robustness check, namely PM_2.5_ and dust.

#### 3.2.1. PM_2.5_

Similar to the main specifications, a stepwise regression method is also used in the robustness check. Columns (1)–(6) in Table 3 provide the estimated results of the two-factor fixed-effect model with only Fintech (in Column (1)) and the complete set of explanatory variables (in Column (6)), respectively.

The results in Table 3 confirm that the key variable of interest, Fintech, has a consistently significant negative effect on the emission level of PM_2.5_ across six different models. The estimates in model 6 show that a one per cent increase in Fintech development will result in a 0.0067-unit drop in local PM_2.5_ emissions on average. This result suggests that the promising effect of Fintech development on lowering SO_2_ emission levels is also reflected in reducing PM_2.5_ emissions. In terms of other covariants, the coefficient of green coverage remains insignificant, the same as urbanization. The number of university students does not significantly impact local PM_2.5_ emissions. One possible explanation is that it is unlikely that the cities dominated by higher education are also dominated by industries that contribute to large-scale PM_2.5_ emissions.

#### 3.2.2. Dust

Table 4 presents the results of our robustness check with the other alternative outcome variable, the dust level. Similarly, a stepwise regression method is employed, and Columns (1)–(6) show the estimated results of the fixed-effect model with Fintech and other gradually added explanatory variables.

The estimated impact of Fintech on local dust levels is consistently significant and negative across six models in Table 4. According to the results of model 6 in Table 4, a one per cent improvement in Fintech can result in a 0.07 per cent reduction in the local dust level, suggesting that more development in Fintech can also result in a significantly lower level of dust in the air. In terms of other explanatory variables, green coverage is not significant in explaining the variation in the dust level, while urbanization leads to a significant increase in the dust level.

Overall, the results shown in Table 3 and Table 4 confirm that Fintech can significantly reduce air pollution, including SO_2_, PM_2.5_, and dust. Hypothesis 1 is thus verified. Fintech uses big data and artificial intelligence to facilitate the green transformation of consumers and SMEs [55]. In the meantime, Fintech can combat environmental pollution and climate change by promoting clean energy trade, improving carbon trading and increasing climate finance flows [30]. For the control variables, FDI significantly negatively affects emissions of all three pollutants, indicating that the “pollution halo” effect of FDI is greater than its “pollution refuge” effect for the sample. One possible explanation is that foreign companies entered China with more advanced and cleaner technologies and management and, as a result, improved the environmental quality of the host country [51,52,53].

### 3.3. Channel Analysis

This section tests two potential mechanisms proposed in Hypothesis 2: green innovation and digital finance. We argue that the Fintech development may have caused a significant reduction in air pollution via green innovation and digital finance in the local region. The model used for the channel analysis is presented as follows:

(2) $$channel_{ct}=c+\alpha Fintech_{ct}+X_{ct}^{\prime }\gamma +\mu_{c}+\mu_{t}+\varepsilon_{ct}$$

(3) $$lnSO_{2}{}_{ct}=c+\theta channel_{ct}+X_{ct}^{\prime }\gamma +\mu_{c}+\mu_{t}+\varepsilon_{ct}$$

where *c* is the constant term; $channel_{ct}$ refers to the measures of green innovation and digital finance of city *c* in year *t*; $ln{\mathrm{SO}}_{2}{}_{ct}$ is the natural logarithm of SO_2_ emissions of city *c* in year *t*; $Fintech_{ct}$ is the Fintech index representing city *c*’s Fintech level in year *t*; $X_{ct}$ contains a set of city-level control variables, including GDP, FDI, number of university students, urbanization, and green coverage; $\mu_{c}$ is the city-fixed effect; and $\mu_{t}$ is the time (year)-fixed effect.

Therefore, Equation (2) estimates the influence of Fintech development on the channel to be tested, while Equation (3) estimates the impact of the potential channel on SO_2_ emissions. If the regression coefficient θ passes the significance test, it shows that the channel we propose has a significant impact on the local SO_2_ emissions. If both α in Equation (2) and θ in Equation (3) pass the significance tests, we argue that Fintech has an impact on the local SO_2_ emissions through the mechanism, meaning the channel effect is established.

This paper proposes two potential channels, namely green patents and digital finance. For the former, we obtain the number of green patents from the China National Intellectual Property Administration and use the natural logarithm of the number. For the latter channel, we use the Digital Financial Inclusion Index from Peking University as the measure. The estimated results of the channel analysis models are presented in Table 5.

Columns (1)–(2) and Columns (3)–(4) in Table 5 show the results of models testing green innovation and digital finance as potential channels, respectively. The Column (1) results suggest that every one per cent increase in local Fintech development will lead to a 0.09 per cent improvement in the number of green patents in that city. The significant estimate confirms that development in Fintech indeed boosts the growth of green innovation. Moreover, the Column (2) results show that every one per cent increase in the number of green patents results in a significant 0.05 per cent drop in the local SO_2_ emissions. In terms of digital finance, similar results are also found in Column (3) and Column (4). Specifically, a one per cent increase in Fintech development leads to 0.0076-unit advancement in digital finance and a one per cent rise in digital finance. Every unit increase in the digital finance index then contributes to a lower SO_2_ emission level by one per cent. Therefore, both green innovation and digital financial mechanisms have been established, and Hypothesis 2 holds.

In brief, Fintech can reduce air pollution by promoting green innovation and digital finance. We believe that Fintech endorses the development of the digital finance that provides more financial support to enterprises. Such support might be particularly critical for energy-intensive and high-polluting enterprises and lay the economic foundation for a green transformation, especially as authorities tighten emission controls.

## 4. Conclusions and Policy Implication

Over the past 40 years of reform and opening up, China’s economy has achieved rapid growth; however, it has also paid the price of environmental pollution. Specifically, the materials used in China‘s major industrial production processes can cause serious environmental pollution, such as steel, cement, coke, etc. In turn, the large-scale use of these materials has led to a substantial increase in the emissions of air pollutants such as carbon dioxide, sulfur dioxide, and nitrogen oxides.

Economically, China faces the challenge of transforming from an extensive growth model to an intensive growth model [56]. Environmentally, China is under pressure to control pollutant emissions and restore a sustainable natural environment. Hence, there is an urgent need for China to explore greener economic development pathways to ensure its people can enjoy a prosperous and healthy natural environment.

Fintech, the combination of finance and technology [12], could be a solution. Technological advancements can change how the financial industry operates. With the evolution of the internet, mobile phones, information technologies and digital technologies [14], Fintech is also embracing its remarkable development. Importantly, Fintech has green attributes and can contribute to green finance and sustainable development [25].

This paper investigates the research question of whether Fintech development has any impact on local air pollution levels and, if yes, whether digital finance and green innovation are the mechanism channels. To answer these two questions, we analyze city-level panel data in China from 2011 to 2017. The Fintech level in each city is measured as the natural logarithm of the number of Baidu News search results. For each city, the search keywords are set in the form of “city name + Fintech-related keyword”, where a set of 48 Fintech-related keywords based on [50] is used. Moreover, this paper replaces the outcome variable with two alternative air pollution indicators to ensure the robustness of the results. The research results show that: (1) The development of Fintech can significantly reduce air pollution. (2) Digital finance and green innovation are two important mechanism channels for Fintech to reduce air pollutant emissions. (3) The robustness check results confirm that the significant and negative effect of Fintech on SO_2_ emissions also apply to the PM_2.5_ emissions and dust levels in the air.

Therefore, this paper proposes several policy recommendations. First, we call on the government to continue to vigorously support the development of Fintech. For example, the government can formulate policies or specific plans to promote investment in Internet and digital information technology and its application in the field of Fintech. Second, the advancement of Fintech and related technologies should be particularly prioritized in less-developed and/or heavily polluted areas. Lastly, the financial industry should be encouraged to strategically optimize services and support enterprises in transitioning to green production. Meanwhile, the government should promote the green transformation of highly polluting industries.

## Figures and Tables

**Table 1:** Descriptive Statistics.

Variable	Description	Obs	Mean	Std. Dev.	Min	Max
SO_2_	Natural logarithm of SO_2_ emissions	1459	10.41	1.13	0.69	13.18
Fintech	Natural logarithm of the number of annual Baidu News search results for “city name + a Fintech-related keyword”	1465	2.70	1.07	0.00	6.01
Green coverage	% of green area in each city	1428	40.38	11.77	0.39	37.66
Urbanization	The land area of municipalities/land area of prefecture-level cities	1461	0.26	0.32	0.00	7.43
FDI	Natural logarithm of FDI	1412	10.49	1.73	1.10	14.94
No. of university students	Natural logarithm of the number of university students	1454	10.82	1.31	6.70	13.88
GDP	City-level gross domestic product value	1460	17,568,123	32,843,303	558,420	3.063 × 10^8^

Table 1 is calculated by the authors.

**Table 2:** Stepwise regression results table of SO_2_.

	(1)	(2)	(3)	(4)	(5)	(6)
Outcome Variable	SO_2_	SO_2_	SO_2_	SO_2_	SO_2_	SO_2_
Fintech	−0.10 ***	−0.09 ***	−0.08 ***	−0.08 ***	−0.07 ***	−0.07 **
	(−3.33)	(−3.07)	(−3.01)	(−3.00)	(−2.67)	(−2.58)
Green coverage		−0.00	−0.00	−0.00	−0.00	−0.00
		(−0.85)	(−0.67)	(−0.65)	(−0.81)	(−0.96)
FDI			−0.06 ***	−0.06 ***	−0.06 ***	−0.05 ***
			(−3.58)	(−3.58)	(−3.27)	(−2.91)
Urbanization				0.03	0.03	0.01
				(1.06)	(1.08)	(0.66)
No. of university students					0.17 ***	0.15 **
					(2.61)	(2.41)
GDP						−0.00 ***
						(−3.92)
Constant	10.71 ***	10.79 ***	11.40 ***	11.39 ***	9.49 ***	9.74 ***
	(132.31)	(71.00)	(49.62)	(49.81)	(12.45)	(13.01)
No. of Obs.	1330	1293	1238	1238	1227	1225
R-square	0.92	0.92	0.93	0.93	0.93	0.93
City FE	YES	YES	YES	YES	YES	YES
Year FE	YES	YES	YES	YES	YES	YES

Table 2 is calculated by the authors. *** represents significant at the level of 1%; ** represents significant at the level of 5%.

**Table 3:** Stepwise regression results table of PM_2.5_.

	(1)	(2)	(3)	(4)	(5)	(6)
Outcome Variable	PM_2.5_	PM_2.5_	PM_2.5_	PM_2.5_	PM_2.5_	PM_2.5_
Fintech	−0.86 ***	−0.81 ***	−0.61 *	−0.61 *	−0.67 **	−0.67 **
	(−3.02)	(−2.78)	(−1.92)	(−1.91)	(−2.08)	(−2.04)
Green coverage		0.02	0.02	0.02	0.02	0.02
		(0.97)	(0.86)	(0.88)	(0.89)	(0.80)
FDI			−0.98 ***	−0.98 ***	−1.00 ***	−0.96 ***
			(−3.97)	(−3.97)	(−4.03)	(−3.82)
Urbanization				0.35	0.35	0.27
				(1.32)	(1.29)	(1.20)
No. of university students					−0.84	−0.93
					(−0.80)	(−0.88)
GDP						−0.00 *
						(−1.77)
Constant	49.89 ***	49.12 ***	59.55 ***	59.43 ***	69.01 ***	70.34 ***
	(63.76)	(41.89)	(21.76)	(21.70)	(6.07)	(6.17)
No. of Obs.	1336	1299	1244	1244	1233	1231
R-square	0.95	0.95	0.95	0.95	0.95	0.95
City FE	YES	YES	YES	YES	YES	YES
Year FE	YES	YES	YES	YES	YES	YES

Table 3 is calculated by the authors. *** represents significant at the level of 1%; ** represents significant at the level of 5%; * represents significant at the level of 10%.

**Table 4:** Stepwise regression results table of dust.

	(1)	(2)	(3)	(4)	(5)	(6)
Outcome Variable	Dust	Dust	Dust	Dust	Dust	Dust
Fintech	−0.10 ***	−0.10 ***	−0.09 **	−0.09 **	−0.07 **	−0.07 **
	(−3.10)	(−2.98)	(−2.48)	(−2.47)	(−2.10)	(−2.10)
Green coverage		−0.00	−0.00	−0.00	−0.00	−0.00
		(−1.18)	(−1.19)	(−1.12)	(−1.32)	(−1.37)
FDI			−0.06 **	−0.06 **	−0.06 **	−0.063 **
			(−2.11)	(−2.12)	(−2.13)	(−2.26)
Urbanization				0.10 **	0.10 **	0.10 **
				(2.49)	(2.35)	(2.43)
No. of uni. students					0.33 ***	0.33 ***
					(2.93)	(2.93)
GDP						0.00
						(0.55)
Constant	10.26 ***	10.38 ***	11.01 ***	10.98 ***	7.34 ***	7.35 ***
	(113.48)	(72.59)	(33.66)	(33.46)	(5.63)	(5.62)
No. of Obs.	1330	1293	1238	1238	1227	1225
R-square	0.88	0.88	0.88	0.88	0.88	0.88
City FE	YES	YES	YES	YES	YES	YES
Year FE	YES	YES	YES	YES	YES	YES

Table 4 is calculated by the authors. *** represents significant at the level of 1%; ** represents significant at the level of 5%.

**Table 5:** Channel effect table.

	(1)	(2)	(3)	(4)
Outcome Variable	Green Patent	SO_2_	Index	SO_2_
Fintech	0.09 **		0.76 **	
	(2.14)		(2.00)	
Green Coverage	0.00	−0.00	0.07 **	−0.00
	(1.14)	(−1.01)	(2.02)	(−0.94)
FDI	−0.01	−0.05 ***	0.57 **	−0.05 ***
	(−0.39)	(−3.11)	(2.45)	(−2.84)
Urbanization	−0.28 ***	0.00	−1.32 ***	0.01
	(−7.44)	(0.05)	(−3.68)	(0.27)
No. of university students	−0.03	0.17 ***	−0.97	0.16 ***
	(−0.20)	(2.67)	(−0.89)	(2.60)
GDP	0.00	−0.00 ***	0.00 ***	−0.00 ***
	(0.96)	(−3.85)	(4.01)	(−3.43)
Green Patent		−0.05 **		
		(−2.15)		
Digital Finance Index				−0.01 **
				(−2.11)
Constant	3.56 **	9.57 ***	148.82 ***	10.35 ***
	(2.42)	(12.99)	(11.96)	(11.55)
No. of Obs.	1231	1225	1231	1225
R-square	0.67	0.93	1.00	0.93
City FE	YES	YES	YES	YES
Year FE	YES	YES	YES	YES

Table 5 is calculated by the authors. *** represents significant at the level of 1%; ** represents significant at the level of 5%.

## Data Availability

No new data were created or analyzed in this study. Data sharing is not applicable to this article.
